# Using microcontrollers and sensors to build an inexpensive CO_2_ control system for growth chambers

**DOI:** 10.1002/aps3.11393

**Published:** 2020-10-14

**Authors:** Haoran Chen, John Markham

**Affiliations:** ^1^ Department of Biological Sciences University of Manitoba Winnipeg Manitoba Canada

**Keywords:** Arduino, CO_2_ concentration, CO_2_ sensor, growth chamber, microcontrollers, valves

## Abstract

**PREMISE:**

A CO_2_ control system is important for investigating how elevated CO_2_ affects plant growth. Our automatic CO_2_ monitoring and control system offers an inexpensive and flexible way to make CO_2_‐enriched environments.

**METHOD AND RESULTS:**

Using microcontrollers paired with non‐dispersive infrared CO_2_ sensors, relays, and valves, we developed a low‐cost system for monitoring and controlling CO_2_ levels in growth chambers.

**CONCLUSIONS:**

Compared with existing commercially available CO_2_ control systems, Arduino‐based microcontrollers offer affordable access to the data logging of CO_2_ levels in growth chambers, thereby reducing budget limitations for creating growth conditions with highly controlled CO_2_ concentrations.

The atmospheric CO_2_ level has increased by 30% over the past 50 years and is predicted to increase up to 800 ppm by 2100 (Thuiller et al., 2005; Lindsey, 2020). Elevated CO_2_ is expected to increase carbon storage and plant productivity due to the enhanced carbon fixation in ecosystems (Drake et al., 2011). In the longer term, rising CO_2_ would increase the carbon : nitrogen ratio within plant tissue and lead to progressive nitrogen limitation in the ecosystem (Luo et al., 2004). Elevated CO_2_ also has a number of direct effects on plant performance, including increasing water use efficiency by increasing biomass accumulation and reducing water loss (Li et al., 2003).

Growth chamber studies have played an important role in examining the effect of CO_2_ levels on plant performance because they can simulate a range of growth conditions and provide a degree of replication and control that is difficult to achieve in the field. Manipulating CO_2_ concentrations in a growth chamber requires a CO_2_ sensor to monitor the concentration of the gas, as well as a system for delivering CO_2_ to the chambers (Tissue and Oechel, 1987). The first CO_2_ control systems relied on infrared CO_2_ analyzers (e.g., the Beckman 864 Process Infrared CO_2_ Analyzer [Beckman Coulter, Brea, California, USA]; Finn and Brun, 1982); however, these analyzers were quite expensive, so CO_2_ control systems typically had to rely on a single sensor to monitor multiple chambers. Some commercially available systems are controlled by a computer via interface cards (e.g., AOP6 and ADC42 [Blue Chip Technology, Chester, United Kingdom]; Barton et al., 1993; Temperton et al., 2003) and can monitor up to 24 chambers. Because earlier infrared sensors required that air samples be desiccated and that samples from each chamber be stabilized before the chamber CO_2_ level could be assessed and adjusted, these systems can be cumbersome to use. A more recent study described a less expensive CO_2_ injection system that was regulated by an infrared gas monitor, where CO_2_ was mixed with ambient air to achieve a target CO_2_ concentration before being added to the growth chambers (Godfree et al., 2011). The system relied on solenoid valves and flow meters to create the target CO_2_ levels. However, its reliance on flow meters caused this system to take a longer time to reach the target CO_2_ level because fresh air and pure CO_2_ must be mixed before being added to the chambers; therefore, the CO_2_ level ranges from 500 to 1000 ppm when the target CO_2_ level is set at 600 ppm. A CO_2_ control system that does not require CO_2_ and fresh air to be mixed before being injected into the growth chamber would provide more stable CO_2_ levels.

Here, we describe a simple, low‐cost CO_2_ control system that uses microcontrollers paired with non‐dispersive infrared (NDIR) CO_2_ sensors to monitor and control the CO_2_ level in growth chambers. Like other infrared sensors, NDIR sensors rely on the principle that a target gas (e.g., CO_2_) absorbs a specific band of infrared light while transmitting other wavelengths of light, thus reducing the energy reaching an infrared light detector (Dinh et al., 2016). A challenge with this technology is the need to reduce interference from non‐target gases, such as water vapor, although recent advances in filtering in NDIR sensors have greatly reduced this interference (Yasuda et al., 2012). NDIR sensing technology has therefore been proven to be durable and stable for detecting CO_2_ in a range of environmental settings; however, because temperature and duration of use can affect sensors (Yasuda et al., 2012), periodic calibration is still essential. Furthermore, the electrical noise associated with growth chambers (especially from lighting systems) can potentially interfere with the communication between a CO_2_ sensor and its data acquisition system. To avoid this, we paired each sensor with a microcontroller in close physical proximity to the sensor and used serial communication, as opposed to a more electrically sensitive digital communication between the sensor and microcontroller. This setup offers an inexpensive and flexible way of making CO_2_‐enriched environments, including the data logging of CO_2_ levels in growth chambers, and can easily be modified to accommodate additional sensors and environmental controls.

## METHODS AND RESULTS

Our system is based on K30 NDIR CO_2_ sensors (Senseair AB, Delsbo, Sweden). They are inexpensive and have integrated calibration and communication circuits (see Table 1 for materials list). They are not sensitive to water vapor, meaning there is no need to use a desiccant to absorb water before sampling. NDIR CO_2_ sensors come in a range of sensitivities and can send digital or analog signals to computer interfaces. The model we used can be calibrated at 0 or 400 ppm CO_2_ by adding a contact switch to its circuit board (CO2meter.com, 2020). The sensors also easily connect to air supply tubing for calibration. Calibration takes approximately 3–5 min, pumping an air stream of 0 or 400 ppm CO_2_ air over the sensor, and then closing the appropriate contact on the sensor circuit board. The sensors’ circuit board and all of the other electrical components of our system have standard 2.5‐mm electrical connector spacings, allowing the wiring to be connected with header pins and connectors (Dupont, JST type, or screw terminals), which are widely available from electronic parts suppliers.

**TABLE 1:** Materials list to build the automatic CO_2_ monitoring and control system.

Materials	Cost (US$)	Model no.	Supplier^a^
CO_2_ sensor	$85	K30	https://www.co2meter.com
Microcontroller^b^	$20	Arduino Uno	https://store.arduino.cc/usa/arduino‐uno‐rev3
Data logging shield	$14	1141	https://www.adafruit.com/
Eight‐channel relay	$12	TS0012	https://www.sunfounder.com
¼‐inch NC gas solenoid valve^b^	$10	2W‐025‐08	
Gas manifold	$100		
2.54‐mm connectors	$10		
22‐gauge wire	$20		
Tank regulator	$200		
Power supplies (9 V^b^, 12 V, 5 V)	$30		
CO_2_ supply tank	$30		

^a^ Items without a supplier are widely available from a number of suppliers.

^b^ One per sensor.

**FIGURE 1:**
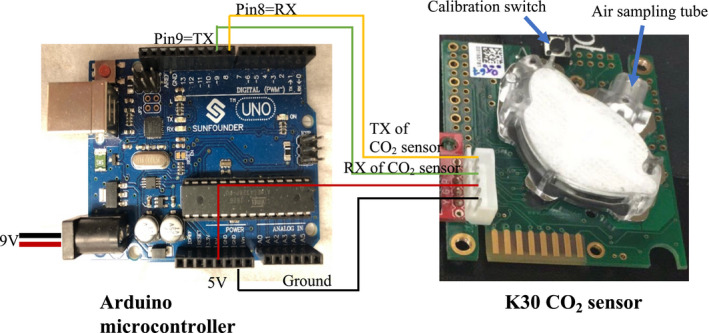
Wiring connections between the K30 CO_2_ sensor (right) and the Arduino Uno–type microcontroller (left) using serial communication. A JST connector was soldered to the K30 circuit board and a calibration switch was soldered to the board to enable the recalibration of the CO_2_ sensor. Calibration gas can be passed through the air sampling tube during calibration. The 5 V and ground pins of the CO_2_ sensor are connected to the corresponding 5 V and ground pins of the microcontrollers (red and black wires, respectively). Pin 8 (RX on the microcontroller) connects to the TX pin on the sensor (yellow wire), and pin 9 (TX on the microcontroller) connects to the RX pins of the CO_2_ sensor (green wire). The data logging shield (not shown) stacks onto the microcontroller. For use, the sensor is placed in an acrylic box with vent holes and mounted on the side of the growth chamber.

**FIGURE 2:**
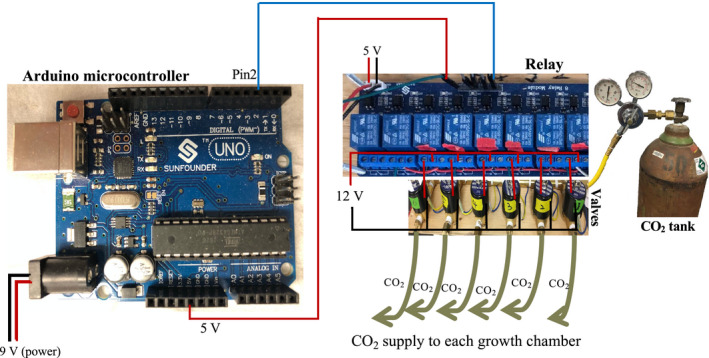
Connections between the Arduino microcontroller and the relays controlling the gas solenoid valves. Each microcontroller (shown with data logging shield removed) is wired to the relay board from its 5 V connection and digital pin (pin 2) to the 5 V connection and corresponding pin controlling a switch on the relay board. The relay board has its own 5 V power supply. The 12 V power supply for each solenoid valve runs through a corresponding relay switch. The CO_2_ tank is connected to gas solenoid valves that are controlled by the Arduino microcontroller and the relays, which regulate the injection of pure CO_2_ into each growth chamber.

**FIGURE 3:**
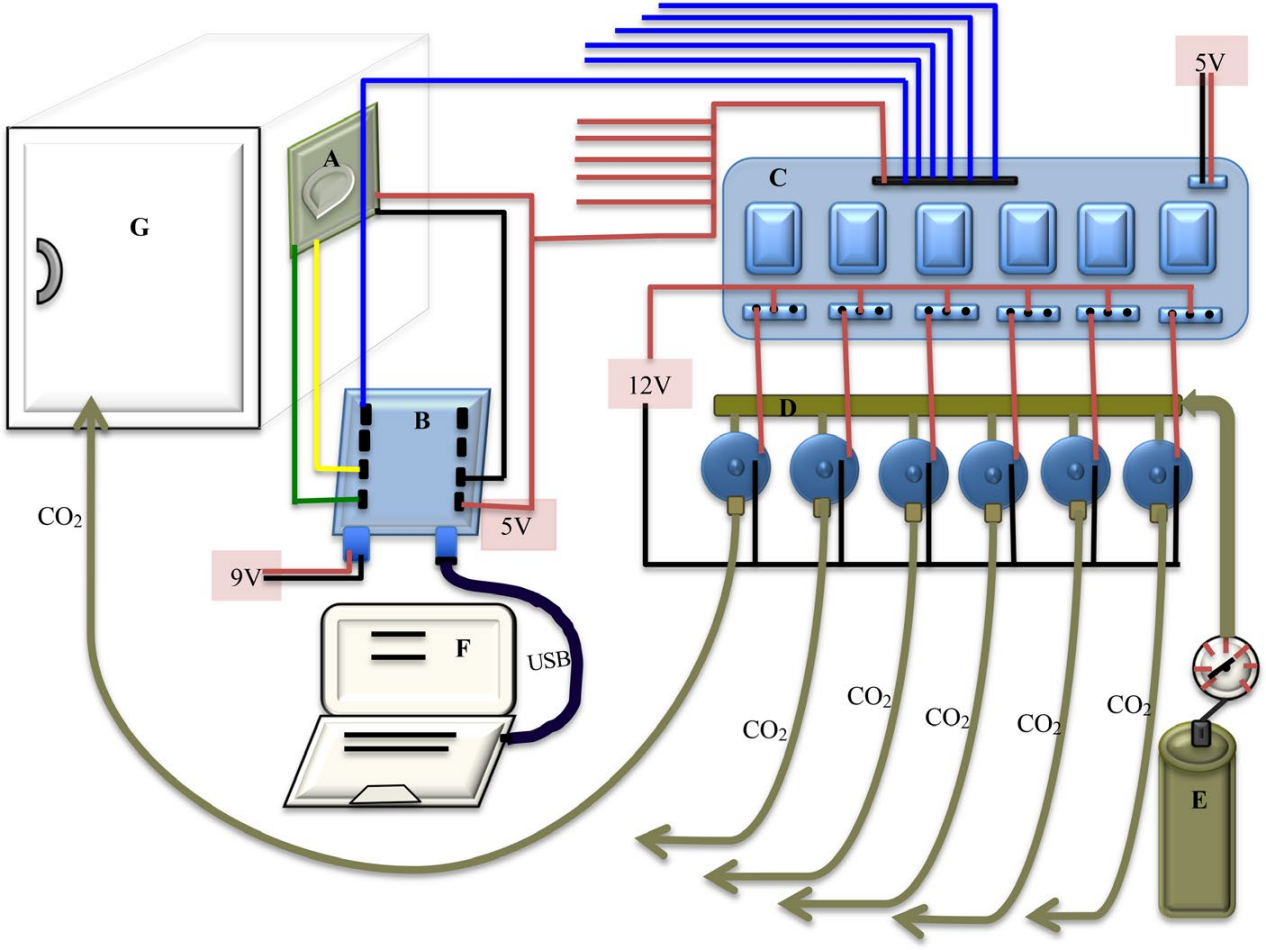
Overall layout of the CO_2_ control system with one growth chamber: CO_2_ sensor (A), Arduino microcontroller (B), relay board (C), gas solenoid valves (D), CO_2_ tank (E), computer (F), and growth chamber (G). Each CO_2_ sensor is attached to an internal wall of the growth chamber and communicates with an individual Arduino microcontroller outside the growth chamber. The extra five red (5 V) and blue (relay signals) wires from the relay board can be connected to five additional microcontroller 5 V pins and digital pins, respectively.

Because growth chambers generate considerable electrical noise, CO_2_ sensors need to be in close proximity to their signal processors to ensure that sensor readings do not degrade. In our system, each CO_2_ sensor is therefore connected to a separate microcontroller mounted on each growth chamber (Fig. 1), which then controls a gas solenoid valve, via a relay switch, on a central manifold connected to a CO_2_ supply tank (Figs. 2, 3). We used Arduino Uno‐type microcontrollers to communicate with the CO_2_ sensors. We used a data logging shield (Adafruit, New York, New York, USA), a real‐time clock, and an SD card socket connected to each Arduino, following the manufacturer’s instructions (Earl, 2013). The large SD cardholder can fit any SD card and store up to 32 GB of information (FAT16 or FAT32 format). The microcontroller can also communicate directly to a computer via a USB or wireless connection, allowing data to be monitored in real time and the microcontroller to be reprogrammed as needed.

The CO_2_ sensors receive their power supply from the microcontroller. Arduino‐type microcontrollers can have a range of voltage inputs, but we found that providing 9 V of power to the microcontroller worked best. If supplied with less than 7 V, the microcontroller and sensor may be unstable and fail to record sensor readings. If using 12 V or higher, the voltage regulator of the microcontroller may overheat. Serial communications between the CO_2_ sensor and the microcontroller require a four‐wire connection (Fig. 1), made via the connectors on the data logging shield stacked on the microcontroller. The sensors can also communicate with the microcontrollers using a digital protocol, which could theoretically allow multiple sensors from many chambers to be connected to a single microcontroller; however, due to the electrical interference in growth chambers, each sensor needs to be placed in close proximity to a microcontroller so serial communication is more stable. The 5 V and ground pins of the sensor are connected to the corresponding 5 V and ground pins of the microcontroller. The transmitting (TX) pin of the sensor is connected to pin 8 (a receiving [RX] pin) of the microcontroller. The RX pin of the sensor is connected to pin 9 of the microcontroller (the microcontroller’s TX pin).

The CO_2_ injection system consists of a compressed CO_2_ gas tank connected to a manifold of solenoid valves activated by electrical relays (Figs. 2, 3). Gas solenoid valves come in a range of voltages, but 12 V models are the most commonly available. Valves with 0.25‐inch connections can be attached to a manifold made of brass tubing and T connectors, which are commonly available from plumbing or compressed air equipment suppliers. The CO_2_ supply tank is connected to the manifold via a compressed air hose with a quick‐release connection, so the tank can be easily replaced when empty. The CO_2_ pressure in the manifold was adjusted to a low level (approximately 10 psi) because very little CO_2_ is needed to increase its concentration in a chamber. Plastic tubing is used to connect the manifold to each growth chamber, and the CO_2_ is released into the chamber at the end opposite the sensor.

Each microcontroller is connected to a relay switch controlling a valve on the CO_2_ injection system (Fig. 2). Relay boards come in various configurations, and it is convenient but not essential to have a relay board with at least as many channels as there are sensors. Our system uses an eight‐channel relay board with its own 5 V power supply; relay boards can be powered by a microcontroller, but this is not recommended due to the relay board’s power consumption. The relay board can be wired such that the relays are in a closed position when not powered, which avoids injecting CO_2_ into a chamber if there is a power interruption. Each microcontroller 5 V pin is connected to the 5 V pin of the relay board. A digital pin on each microcontroller is connected with a pin on the relay board, with each controlling a different relay switch on the board.

**FIGURE 4:**
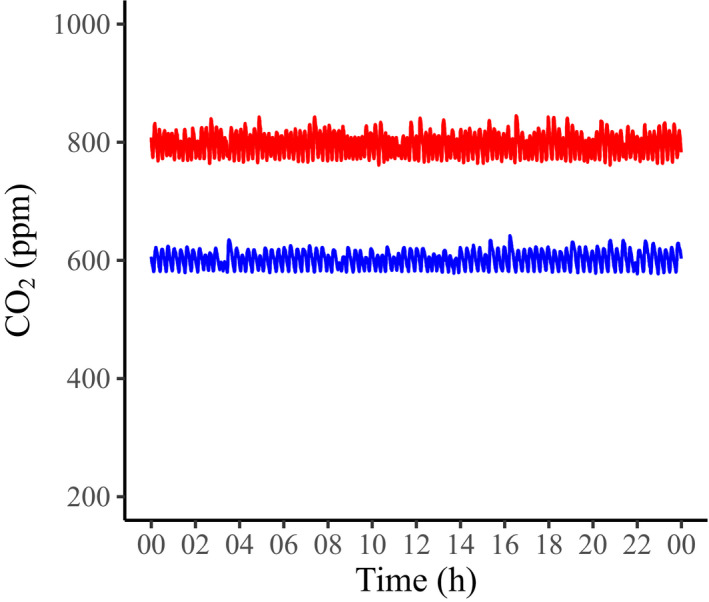
An example of CO_2_ levels (600 ppm and 800 ppm target levels) in the growth chambers recorded over a 24‐h period.

The microcontroller is programmed using Arduino software (https://www.arduino.cc/en/main/software). The code provided here (Appendix S1) is an example of a chamber set to achieve an 800 ppm CO_2_ concentration (Fig. 4). The chamber numbers, CO_2_ levels, monitoring time, valve open time, etc. can be edited within the code based on the experimental conditions. Even a brief injection of CO_2_ will cause a rapid increase in the CO_2_ concentration in the chamber; thus, the CO_2_ level that opens the valve must be set below the desired chamber CO_2_ concentration and the valve should open for a short enough period to result in a CO_2_ increase to near the desired concentration. In this example, the CO_2_ sensors monitor the CO_2_ level every 30 s. If the CO_2_ level in a chamber drops below the set value, the corresponding gas valve is set to open for 0.2 s and will not reopen for 30 s, allowing the air to circulate in the chamber before adding more CO_2_. We tested the system using Conviron A1000 chambers (Conviron, Winnipeg, Canada), which have a 1000‐L volume and were set at 24°C and ca. 36% humidity. The growth chambers had their external air supply valves closed and these were also covered in cellophane tape to reduce air leakage. An internal fan provides rapid air movement within the chamber.

Figure 4 provides an example of the CO_2_ concentration in chambers set to 600 ppm and 800 ppm CO_2_ over a 24‐h period. It took approximately 3 min for the chamber CO_2_ level to increase from the ambient concentration to reach the target CO_2_ level of 600 ppm. To achieve the desired CO_2_ level of 600 ppm, the valve was set to open at 585 ppm and stay open for 0.15 s. This resulted in ca. 45 mL of CO_2_ gas at atmospheric pressure being added to the chamber (i.e., 0.0045% of the volume of the chamber) each time the valve opened, and therefore had a negligible effect on other parameters of the chamber air (e.g., humidity and pressure). During a 24‐h period, the CO_2_ concentration ranged from 577 ppm to 642 ppm in the chamber, with a mean of 602.3 ppm and a standard deviation of 13.6 ppm, resulting in a 2% coefficient of variation. All of the readings were within ±7.0% of the target CO_2_ level. In order to achieve a CO_2_ concentration of 800 ppm, the valve was set to open at 780 ppm and stay open for 0.2 s. The CO_2_ level ranged from 761 ppm to 845 ppm, with a mean of 798 ppm and a standard deviation of 18.3 ppm. All of the readings fell within ±5.6% of the target CO_2_ level. Although the absolute value of the standard deviation was higher at this high CO_2_ target, the coefficient of variation was 2%, the same as it was for 600 ppm. In contrast, the CO_2_ control system described by Godfree et al. (2011) had a coefficient of variation of 15% in its CO_2_ level. In our laboratory, we have run growth experiments using this system for six chambers simultaneously. The use of larger chambers or chambers with less air movement will require adjustments in the air sampling frequencies and the length of time for which the CO_2_ supply remains open. These are easily adjusted in the microcontroller code, and the results can be viewed in real time by connecting the microcontroller to a computer. It is also relatively straightforward to add a display to the microcontroller to view the chamber CO_2_ levels (see, for example, https://www.arduino.cc/en/Tutorial/LiquidCrystalDisplay [accessed 8 September 2020]). Because the NDIR sensors can lose accuracy over time (Yasuda et al., 2012), calibration should be carried out periodically (CO2meter.com, 2020). Although the growth chambers in this study control several environmental conditions, Arduino‐type microcontrollers are compatible with a number of robust sensors (e.g., temperature, humidity, soil water content), allowing the user to easily keep track of and log various environmental conditions. The relays used to control the solenoid valves can also be used as switches for any growth‐related equipment, opening a range of possibilities for adding new functionality to growth experiments.

## CONCLUSIONS

Our CO_2_ control system gives researchers access to a low‐cost setup for monitoring and controlling CO_2_ levels in controlled environmental conditions. The CO_2_ control system can be run in multiple chambers simultaneously, and we provide a versatile code for the CO_2_ control system, which can also be used in other areas (e.g., monitoring and controlling temperature, humidity, and soil moisture). The system can be easily adapted for different experimental conditions, and its low cost allows the replication of conditions to be achieved for more statistically robust growth experiments.

## AUTHOR CONTRIBUTIONS

H.C. designed and executed this work under the guidance of J.M. H.C. wrote the article, and J.M. was involved in the editing and correcting of the manuscript. Both authors approved the final manuscript.

## Supporting information


**APPENDIX S1.** Arduino code for a growth chamber CO_2_ control system.
