# Supplementary material for: Using microcontrollers and sensors to build an inexpensive CO2 control system for growth chambers
Source: Appl Plant Sci. 2020 Oct 14;8(10):e11393. doi: 10.1002/aps3.11393 (PMC7598886; doi:10.1002/aps3.11393)
Supplement: Supplementary file 1 — APPENDIX S1. Arduino code for a growth chamber CO2 control system. [file APS3-8-e11393-s001.docx]

**APPENDIX S1.** Arduino code for a growth chamber CO_2_ control system.

/*

Code for a growth chamber CO2 control system

This code includes: (1) communication between sensors and microcontrollers; (2) setting up the data logging clock; (3) writing data to the SD card; (4) communication between microcontrollers and relays.

The code for serial communications between the CO2 sensor and Arduino was downloaded from <https://www.co2meter.com/collections/sensors/products/k-30-co2-sensor-module>

The connections between the microcontroller and sensors are as follows:

black wires = ground pin of Arduino connected to ground pin of CO2 sensor

red wires = 5 V pin of Arduino connected to 5 V pin of CO2 sensor

yellow wires = pin8 (defined as RX) of the Arduino connected to TX pin of CO2 sensor

green wires = pin9 (defined as TX) of Arduino connected to RX of CO2 sensor

**Values in bold can be adjusted for different CO2 levels, injections times, and periods between sensor measurements.**

*/

#include "kSeries.h" // Used for communicating between the K-series CO2 sensor and the // microcontroller using a Serial signal

/*

In order to check what time of day the data was logged, the code for the real-time clock (RTC) was downloaded from <https://learn.adafruit.com/adafruit-data-logger-shield/using-the-real-time-clock>

*/

// Date and time functions using a PCF8523 RTC connected via I2C and Wire lib

#include <Wire.h>

#include "RTClib.h"

/*

In order to read and write data to and from the SD card file, the code was downloaded from <https://learn.adafruit.com/adafruit-data-logger-shield/for-the-mega-and-leonardo>.

*/

#include <SPI.h>

#include <SD.h>

RTC_PCF8523 rtc; // Define the Real Time Clock object

char daysOfTheWeek[7][12] = {"Sunday", "Monday", "Tuesday", "Wednesday", "Thursday", "Friday", "Saturday"};

// set up variables using the SD utility library functions:

Sd2Card card;

SdVolume volume;

SdFile root;

// Adafruit SD shields and modules: pin 10

// For the data logging shield, we use digital pin10 for the SD CS line

const int chipSelect = 10;

const char* logfile = "chamber1.csv"; // Define the file name and format

// Define variable to turn the CO2 relay on and off.

#define RELAY_ON 0

#define RELAY_OFF 1

// create a variable for each relay. Each relay is associated with a different digital pin

// In this example digital pin 2 is connected to the relay

#define Relay_1 2 // Arduino Digital I/O (digitalRead and digitalWrite) pin number.

kSeries Sensor1(8, 9); // Initialize a kSeries Sensor with pin 8 as Rx and 9 as Tx

// In this example we are calling the sensor “Sensor1”

void setup()

{

// Open serial communications and wait for port to open:

Serial.begin(9600);

while (!Serial) {

; // wait for serial port to connect. Needed for the arduino native USB port connection

}

Serial.println("AN-216 Example 2: uses the kSeries.h library");

//-------(Initialize Pins so relays are inactive at reset)----

digitalWrite(Relay_1, RELAY_OFF);

//---( THEN set pins as outputs )----

pinMode(Relay_1, OUTPUT);

if (! rtc.begin()) {

Serial.println("Couldn't find RTC");

while (1);

}

// If you want to reset the clock, uncomment this line.

// rtc.adjust(DateTime(F(__DATE__), F(__TIME__)));

if (! rtc.initialized()) {

rtc.adjust(DateTime(F(__DATE__), F(__TIME__)));

Serial.println("RTC is NOT running!");

// The following line sets the RTC to the date & time this sketch was compiled

// rtc.adjust(DateTime(F(__DATE__), F(__TIME__)));

// This line sets the RTC with an explicit date & time, for example to set

// January 21, 2014 at 3am you would call:

// rtc.adjust(DateTime(2017, 7, 26, 15, 03, 0));

} else {

Serial.println("Clock already initialized");

}

Serial.print("\nInitializing SD card...");

if (!SD.begin(10)) {

Serial.println("initialization failed!");

return;

}

Serial.println("initialization done.");

// If you want to empty the file, uncomment this.

// SD.remove(“Chamber1.csv”);

// Read existing lines in the file.

File myFile = SD.open(logfile);

if (myFile) {

Serial.print("Writing contents of: ");

Serial.println(logfile);

// Read from the file until there's nothing else in it:

while (myFile.available()) {

Serial.write(myFile.read());

}

Serial.println("Finished writing contents of chamber1.csv");

// close the file:

myFile.close();

} else {

// If the file didn't open, print an error:

Serial.print("error opening: ");

Serial.println(logfile);

}

delay(100); // Nothing will happen for this time to check that all relays are inactive

}

void loop() {

File myFile = SD.open(logfile, FILE_WRITE); // Open Chamber1.csv on the SD card

// as a file to write to

if (myFile) {

// Get current time

DateTime now = rtc.now();

// Print the date to Serial and myFile

// Date format: <year>/<month>/<day> <hour>:<minute>:<second>

Serial.print(now.year(), DEC);

Serial.print('/');

Serial.print(now.month(), DEC);

Serial.print('/');

Serial.print(now.day(), DEC);

Serial.print(' ');

Serial.print(now.hour(), DEC);

Serial.print(':');

Serial.print(now.minute(), DEC);

Serial.print(':');

Serial.print(now.second(), DEC);

Serial.print(", ");

myFile.print(now.year(), DEC);

myFile.print('/');

myFile.print(now.month(), DEC);

myFile.print('/');

myFile.print(now.day(), DEC);

myFile.print(' ');

myFile.print(now.hour(), DEC);

myFile.print(':');

myFile.print(now.minute(), DEC);

myFile.print(':');

myFile.print(now.second(), DEC);

myFile.print(", ");

double co2 = Sensor1.getCO2('p'); // Returns CO2 value in ppm ('p') or percent ('%')

if (co2_ppm > 0){

Serial.print("Sensor1, ");

Serial.print(co2_ppm);

Serial.print(", ");

myFile.print("Sensor1, ");

myFile.print(co2_ppm);

myFile.print(", ");

} else {

Serial.print("Failure: Sensor1,");

myFile.print("Failure: Sensor1,");

}

/*. Set the CO2 level that opens the valve and the time it remains open. In this example, we want to achieve a CO2 level of 800 ppm. The valve is therefore set to open at 780 ppm and stay open for 200 milliseconds

*/

if (co2_ppm < **780** && co2_ppm > 0){

// Open valve if CO2 is too low and there is a CO2 reading

digitalWrite(Relay_1, RELAY_ON);

Serial.print("valve 1 on,");

delay(**200**); // Open valve for 200 milliseconds) if the CO2 level is below

// the set point

digitalWrite(Relay_1, RELAY_OFF);

} else {

digitalWrite(Relay_1, RELAY_OFF);

Serial.print("valve 1 off, ");

}

Serial.println();

myFile.println();

}

myFile.close();

delay(**30000**); // Wait 30 seconds for the air to circulate in the growth chamber and

// check the level again. This delay depends on how quickly the added

// CO2 circulates in the chamber.
